# Autoregulation assessment by direct visualisation of pial arterial blood flow in the piglet brain

**DOI:** 10.1038/s41598-019-50046-x

**Published:** 2019-09-16

**Authors:** S. P. Klein, V. De Sloovere, G. Meyfroidt, B. Depreitere

**Affiliations:** 1 Department of Neurosurgery, University Hospitals Leuven, Leuven, Belgium; 2 Department of Anesthesiology, University Hospitals Leuven, Leuven, Belgium; 3 Department of Intensive Care Medicine, University Hospitals Leuven, Leuven, Belgium

**Keywords:** Neuro-vascular interactions, Neurophysiology, Brain injuries, Neurovascular disorders

## Abstract

Impairment of cerebrovascular autoregulation (CAR) is common after brain injury, although the pathophysiology remains elusive. The mechanisms of vascular dysregulation, their impact on brain function, and potential therapeutic implications are still incompletely understood. Clinical assessment of CAR remains challenging. Observational studies suggest that CAR impairment is associated with worse outcomes, and that optimization of cerebral blood flow (CBF) by individual arterial blood pressure (ABP) targets could potentially improve outcome. We present a porcine closed cranial window model that measures the hemodynamic response of pial arterioles, the main site of CBF control, based on changes in their diameter and red blood cell velocity. This quantitative direct CAR assessment is compared to laser Doppler flow (LDF). CAR breakpoints are determined by segmented regression analysis and validated using LDF and brain tissue oxygen pressure. Using a standardized cortical impact, CAR impairment in traumatic brain injury can be studied using our method of combining pial arteriolar diameter and RBC velocity to quantify RBC flux in a large animal model. The model has numerous potential applications to investigate CAR physiology and pathophysiology of CAR impairment after brain injury, the impact of therapeutic interventions, drugs, and other confounders, or to develop personalized ABP management strategies.

## Introduction

Cerebrovascular autoregulation (CAR) protects the brain against changes in cerebral perfusion pressure (CPP) by actively adjusting the vascular resistance to ensure a steady cerebral blood flow (CBF).

The role and implications of impaired CAR are increasingly being recognized in the pathophysiology of acute brain injuries such as traumatic brain injury (TBI), stroke, subarachnoid haemorrhage (SAH), or prematurity-related intracranial haemorrhage (ICH), but also in chronic neurological conditions such as vascular dementia or Alzheimer’s disease^1,2^. However, the mechanisms underlying efficient and deficient CAR and implications for management in situations of acute brain injury remain speculative so far. Insights have been continuously evolving, giving rise to potentially promising strategies^2–4^. Patients with impaired CAR are highly dependent on adequate cerebral perfusion pressure to maintain adequate CBF. Real-time monitoring of the state of cerebral autoregulation could open the door for individually tailored therapeutic manipulation of arterial blood pressure in patients at risk for secondary brain injury^5^. Observational studies suggest a positive association between proximity of actual CPP to the calculated ‘optimal CPP’ and clinical outcome in TBI patients^6^. Several methods for the clinical assessment of CAR have been developed but their relation to the actual physiological phenomenon of CAR remains unclear. All mathematical methods rely on assumptions and premises that require thorough understanding of the mechanisms underlying CAR. In general, there is disagreement between different metrics of CAR, reflecting a relatively large degree of uncertainty and issues of construct validity^7,8^. The main limitation for all analytical approaches is the lack of a ‘gold standard’ method to quantify CAR.

Pial arterioles on the cerebral cortex control the largest proportion of change in vascular resistance, and as such are the main site of CBF control. Therefore, they play a key role in the protective autoregulation of CBF and also in neurovascular coupling^2^. The use of a relevant animal model to study pial arteriolar regulation of CBF can allow translation of basic scientific advancements into clinical practice.

In order to assess pial arteriolar blood flow as adequate as possible, a combination of two measurements, the vessel diameter as well as the red blood cell (RBC) velocity to calculate RBC flux, is necessary^9,10^. The use of one of these parameters in isolation has been proven insufficient, particularly when blood flow is disturbed and red blood cell velocity and lumen diameter can change independently from each other^10,11^. Rodents models to study the vasodynamics of pial arteriolar flow have been developed, and use confocal or two-photon microscopy measurements of vessel diameters and RBC velocity. However, the translation of these techniques to large animal models is hindered by technical difficulties. The porcine animal model is often preferred over rodent models, specifically in the field of neuroscience because of the similarities of the porcine brain to the human brain with regards to anatomy, growth, and development. The porcine animal model has the additional advantage of being closer to the human cardiorespiratory physiology^12–14^. The larger size of the animal permits handling and catheterization that enable adequate physiological monitoring, with techniques and devices comparable to human patients in the intensive care unit.

We provide a detailed description of a porcine closed cranial window model allowing direct visualization of the cortical microvasculature and enabling the quantitative study of blood flow changes in pial arterioles based on vessel diameter and RBC velocity. We hypothesize that this model can allow for measurement of the hemodynamic response and limits of CAR in pial arterioles in physiological and pathological conditions. We have aimed at validating pial arteriolar blood flow measurements using laser Doppler flowmetry and brain tissue oxygenation measurements during gradually induced non-pharmacological hypo- and hypertension. In addition, we have investigated the feasibility of measurement of the pial arteriolar hemodynamic response using our method in TBI using a controlled cortical impact. A controlled cortical impact (CCI) in a porcine model generates a reproducible injury with pathological features similar to human TBI^14,15^.

## Results

### Arteriolar flux measurement

**Figure 1:**
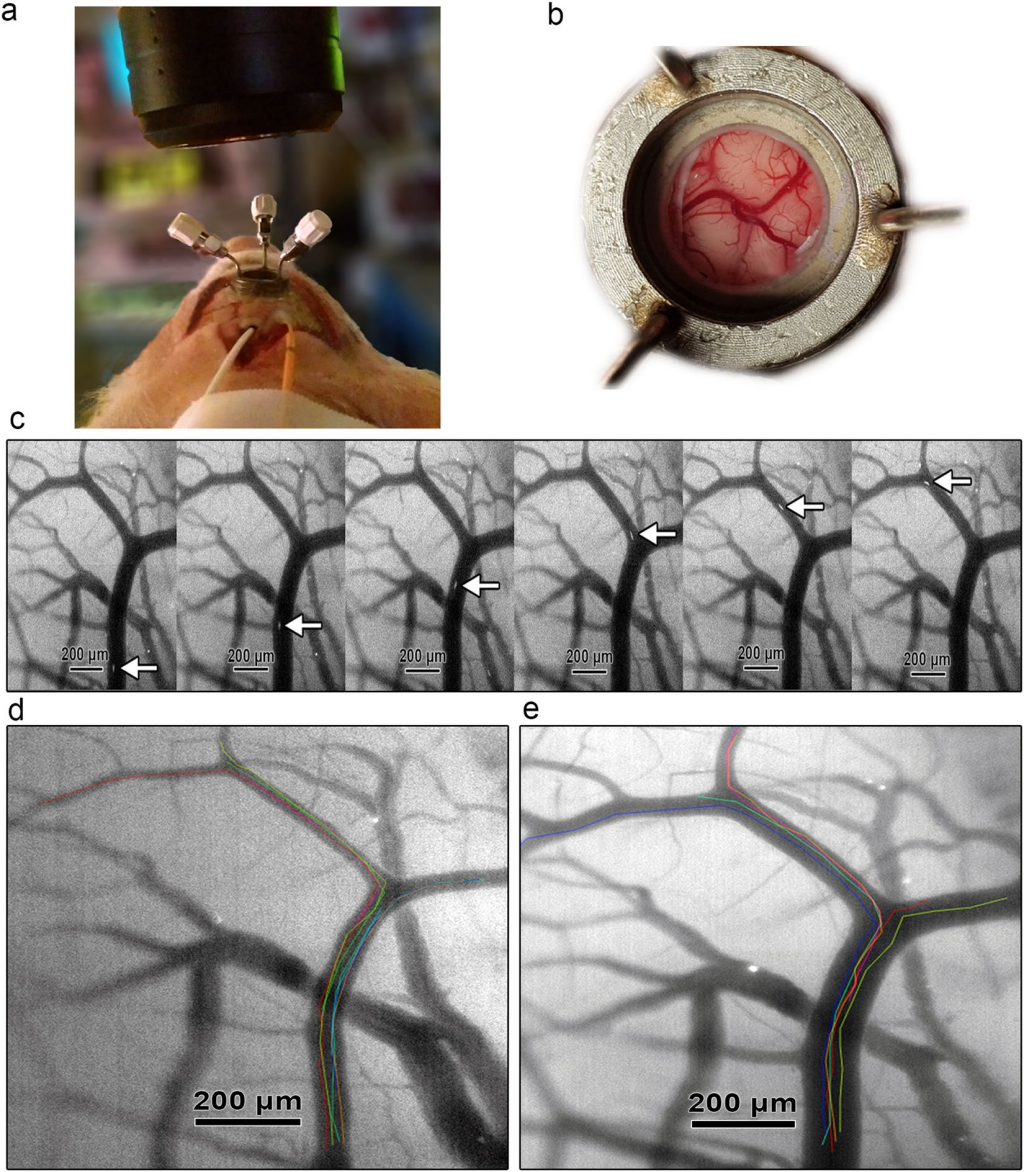
Illustration of the *in vivo* measurement of pial arteriolar RBC flux. (**a**) Microscope positioned over the closed cranial window. Cortical LDF probe (white) and intraparenchymal ICP-PbtO_2_ probe (orange) placed ipsilateral immediately behind the cranial window. (**b**) Overview of cortical vessels as seen through the closed cranial window. (**c**) Fluorescent-labeled RBC moving through a pial arteriole at 200 frames-per-second. (**d**) Baseline visualization of pial arterioles and individual RBC tracks. Individual RBC tracks are superimposed on the original image in different colors. (**e**) Vasodilation of pial arterioles and individual RBC tracks during induced hypotension. Individual RBC tracks are superimposed on the original image in different colors.

To measure *in vivo* RBC flux through pial arterioles we performed high-speed imaging at 170 to 200 frames-per-second of pial arterioles and fluorescent RBCs through a closed cranial window. A fraction of 4–7% of total circulating RBCs was fluorescently labeled with carboxyfluorescein diacetate succinimidyl ester (CFSE), an intracellular fluorescent probe not influencing RBC rheology. Selective GFP fluorescent filter illumination produces negative contrast delineation of the pial vessels due to autofluorescence of the brain parenchyma allowing vessel diameter measurements. Arterioles with a diameter of 20 µm and higher could be visualized. An individual movement track was created per fluorescent RBC and average path speed was calculated for each track. Per arteriole, an overall average of RBC path speeds was calculated for each time point. Diameter measurements were performed for each time point. Under the assumption of steady-state laminar flow in a cylindrical vessel, the Hagen-Poiseuille law was used to define the average volumetric RBC flux based on RBC velocity and lumen diameter measured in a single vessel^16,17^. Repeated imaging could be performed to measure differences in RBC velocity and arteriolar diameter. An illustration of a view through the closed cranial window, RBCs moving through a pial arteriole and RBC tracking is provided in Fig. 1 and Video 1.

**Figure 2:**
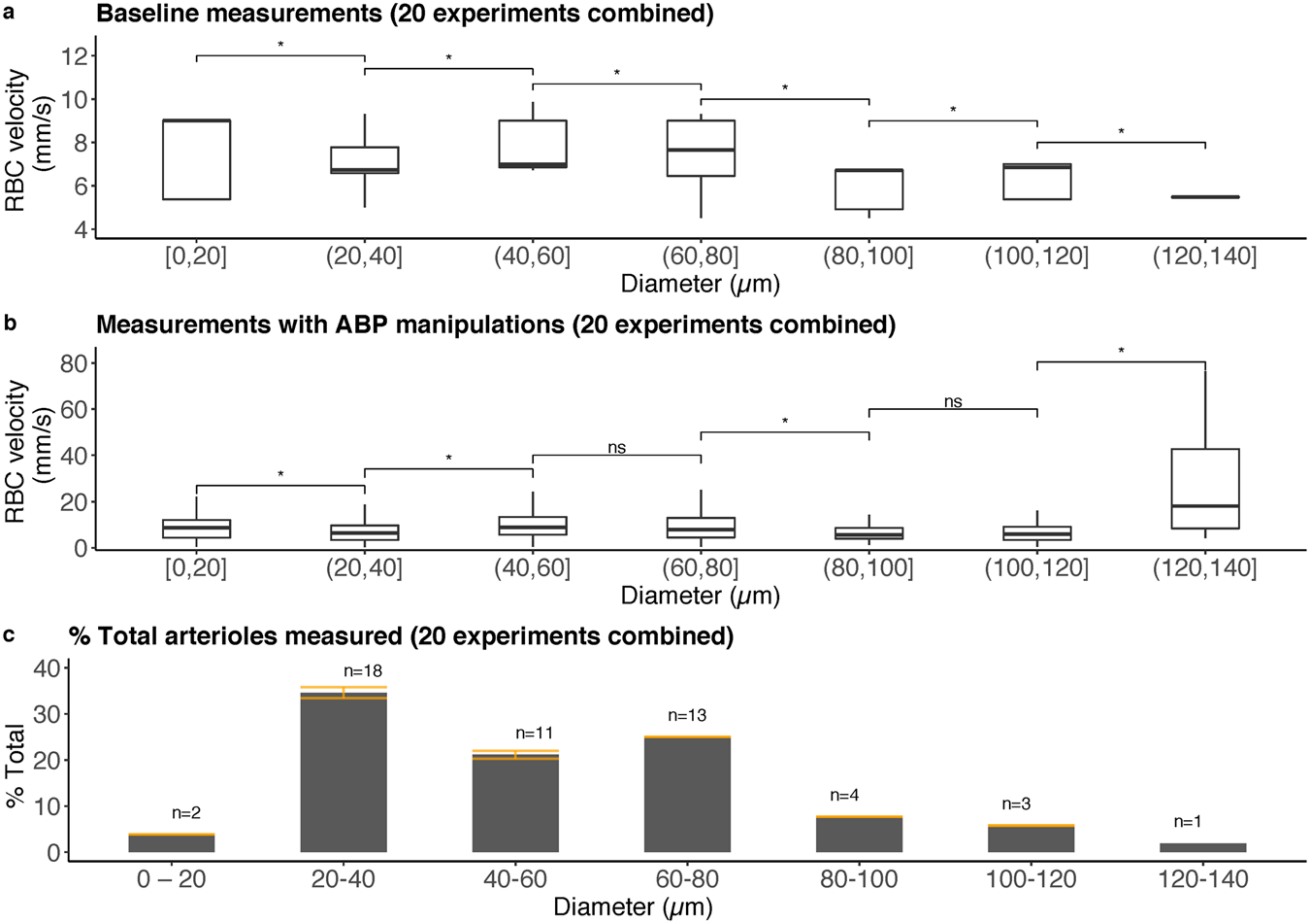
Overview of RBC velocity and pial arteriolar diameters measured in 20 experiments combined (10 hypotensive and 10 hypertensive). A total of 52 randomly selected arterioles were studied with an average of 7.2 RBC tracks per arteriole (SD 3). (**a**) Baseline RBC velocity plotted as a function of pial arteriolar diameter in 20 experiments combined. (**b**) Measurements with ABP manipulations of RBC velocity plotted as a function of pial arteriolar diameter in 20 experiments combined. A significant increase in RBC velocity in diameter category 120–140 μm is the result of hypertensive forced vasodilation. (**c**) Histogram of baseline pial arteriolar diameter category of randomly selected arterioles in the 20 experiments plotted as a percentage of total pial arterioles studied, demonstrating the range of pial arteriolar diameters that were studied. Error bars in orange demonstrate the standard deviation per diameter category. The total number of arterioles (n) with baseline diameter within the diameter category is shown above each bar. Kruskal-Wallis test for analysis of differences between groups (ns: p > 0.05; *p < 0.05).

Using pial arteriolar diameter and RBC velocity, the model is able to estimate dynamic blood flow changes in individual pial arterioles based on RBC flux. For this study, we randomly selected up to 3 arterioles per cranial window experiment ranging from 20 to 140 μm for further analysis. Figure 2 demonstrates RBC velocities and pial arteriolar diameters of 52 randomly chosen arterioles that were studied in 20 experiments (10 hypotensive and 10 hypertensive) during baseline conditions (panel a) and during ABP manipulations (panel b). An average of 7.2 RBC tracks per arteriole (SD 3) was measured.

### Comparison of cranial window derived RBC flux to laser Doppler flowmetry

Laser Doppler flowmetry (LDF) is a well-established tool for continuously monitoring dynamic regional changes of CBF, however lacking the spatial resolution to resolve (patho)physiological responses of individual vessels^18–22^.

We compared changes in arteriolar RBC flux measured through the cranial window to LDF flux changes 1 cm behind the cranial window. A total of 52 arterioles were studied during non-pharmacological manipulation of arterial blood pressure in 10 hypertensive and 10 hypotensive experiments.

Mean baseline pial arteriolar diameter was 54 µm (range 16–123 µm). Pial arteriolar diameter could explain a median of 34% variability in LDF flux by linear regression. RBC velocity could explain a median of 71% of variability in LDF flux. Pial arteriolar diameter and RBC velocity combined to calculate pial arteriolar RBC flux could explain a median of 81% variability in LDF flux (Table 1). We demonstrated a relatively good quantitative assessment of LDF changes by pial arteriolar RBC flux. The main determinant in the prediction of LDF by pial arteriolar RBC flux changes was RBC velocity. LDF changes were not accurately predicted by pial arteriolar diameter changes.

**Table 1:** Goodness of fit for three linear regression models with LDF flux as dependent variable and pial arteriolar diameter or RBC velocity or RBC flux as independent variable using average measures for 5-mmHg CPP bins.

	N Arterioles	Pial Arteriolar Diameter (Median)	Diameter R^2^ (Median)	RBC Velocity R^2^ (Median)	RBC Flux R^2^ (Median)
Hypotension	26	42 µm	0.09	0.61	0.72
Hypertension	26	48 µm	0.60	0.75	0.83
All Combined	52	45 µm	0.34	0.71	0.81

The coefficient of determination (R^2^) is the proportion of variance in LDF flux that is explained by pial arteriolar diameter, RBC velocity or RBC flux.

### Cerebrovascular autoregulation breakpoint comparison between RBC flux, LDF flux, and brain tissue oxygen pressure

**Figure 3:**
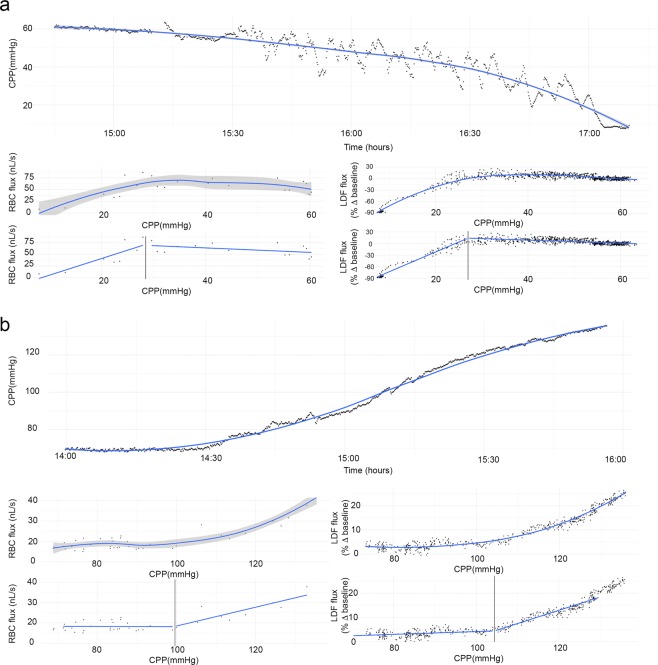
Demonstration of a representative non-pharmacological arterial blood pressure manipulation in 2 animals: hypotension (**a**) and hypertension (**b**). The top panel of a and b shows cerebral perfusion pressure as a function of time. On the left, RBC flux as a function of CPP is shown with a second plot below demonstrating the segmented regression method for breakpoint estimation (vertical line delineates the breakpoint). On the right, LDF as a function of CPP is shown with a second plot demonstrating the segmented regression method for breakpoint estimation. Grey shading represents the standard error.

**Figure 4:**
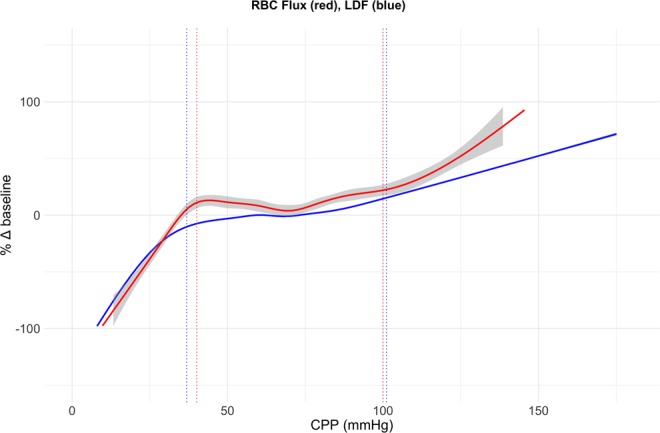
Autoregulation curve Combined changes in LDF (blue) and RBC flux (red) for 10 hypotensive and 10 hypertensive experiments plotted against CPP. Grey shading represents the standard error (for the LDF data curve there was no visible standard error). Dotted lines show the mean lower and upper limits of autoregulation determined by segmented regression analysis of individual experiments as described in Table 2. Differences between breakpoints were not statistically significant for the lower (hypotensive experiments, p = 0.25) and upper (hypertensive experiments, p = 0.72) limits of autoregulation.

CAR breakpoints determined by segmented regression using pial arteriolar RBC flux and cortical LDF flux measurements as a function of CPP are summarized in Table 2. The assumption of normality was confirmed using Shapiro-Wilk test at the level of significance 0.05 and difference between breakpoints in hypotensive (p = 0.25) and hypertensive (p = 0.72) experiments was not significant using paired samples t-test. An illustration of the breakpoint estimation method to determine the limits of CAR is given in Fig. 3. Mean differences between pial arteriolar RBC flux and cortical LDF flux autoregulation breakpoints were 3.2 mmHg (SD 8.3) for the hypotensive experiments and −1.1 mmHg (SD 9.2) for the hypertensive experiments. Between-subject changes in LDF and RBC flux plotted against CPP for the combined group of 10 hypotensive and 10 hypertensive experiments shows a similar pressure – flow relationship as put forward by Lassen *et al*. in 1959 (Fig. 4)^23^. A very strong intraclass correlation and coefficient of determination was found between breakpoints determined by pial arteriolar RBC flux, LDF and brain tissue oxygenation (Table 3). We demonstrate that the limits of CAR using RBC flux in pial arterioles are in agreement with LDF and brain tissue oxygenation (PbtO_2_) estimated limits of CAR.

**Table 2:** Limits of cerebrovascular autoregulation determined by breakpoint segmented regression analysis for 10 hypotensive and 10 hypertensive experiments.

Hypotension				Hypertension			
Experiment	Breakpoint RBC flux (mmHg)	Breakpoint LDF (mmHg)	Difference between breakpoints (mmHg)	Experiment	Breakpoint RBC flux (mmHg)	Breakpoint LDF (mmHg)	Difference between breakpoints (mmHg)
Hypotension 1	37.0	37.8	−0.8	Hypertension 1	89.6	91.8	−2.2
Hypotension 2	41.1	35.9	5.2	Hypertension 2	93.2	100.0	−6.8
Hypotension 3	50.6	56.7	−6.1	Hypertension 3	70,0	70.01	−0.01
Hypotension 4	34.4	37.0	−2.6	Hypertension 4	114.1	90.7	23.4
Hypotension 5	57.0	49.4	7.6	Hypertension 5	82.9	82.4	0.5
Hypotension 6	33.4	38.0	−4.6	Hypertension 6	117.3	123.6	−6.3
Hypotension 7	45.0	22.3	22.7	Hypertension 7	108.5	109.3	−0.8
Hypotension 8	28.0	27.0	1.0	Hypertension 8	97.9	107.8	−9.9
Hypotension 9	36.2	30.0	6.2	Hypertension 9	112.7	116.5	−3.8
Hypotension 10	38.0	34.4	3.6	Hypertension 10	113.5	118.5	−5,0
**Mean (SD)**	**40.1 (8.7)**	**36.9 (10.1)**	**3.2 (8.3)**	**Mean (SD)**	**99.9 (15.9)**	**101.1 (17.2)**	**−1.1 (9.2)**

Differences between breakpoints were not significant using paired samples t-test in the hypotension (p = 0.25) and hypertension (p = 0.72) groups.

**Table 3:** Intraclass correlation (ICC, two-way random effects, absolute agreement) between autoregulation breakpoints determined by RBC flux, LDF flux, and PbtO_2_.

	ICC (95% CI)	R^2^	p-value
RBC flux - LDF flux breakpoints	0.97 (0.93–0.99)	0.94	<0.0001
RBC flux - PbtO_2_ breakpoints	0.95 (0.86–0.98)	0.89	<0.0001
PbtO_2_ - LDF flux breakpoints	0.95 (0.88–0.98)	0.91	<0.0001

Coefficient of determination (R^2^) between autoregulation breakpoints determined by linear regression and significance (p-value).

### *In vivo* RBC flux measurement in a porcine model of traumatic brain injury

**Figure 5:**
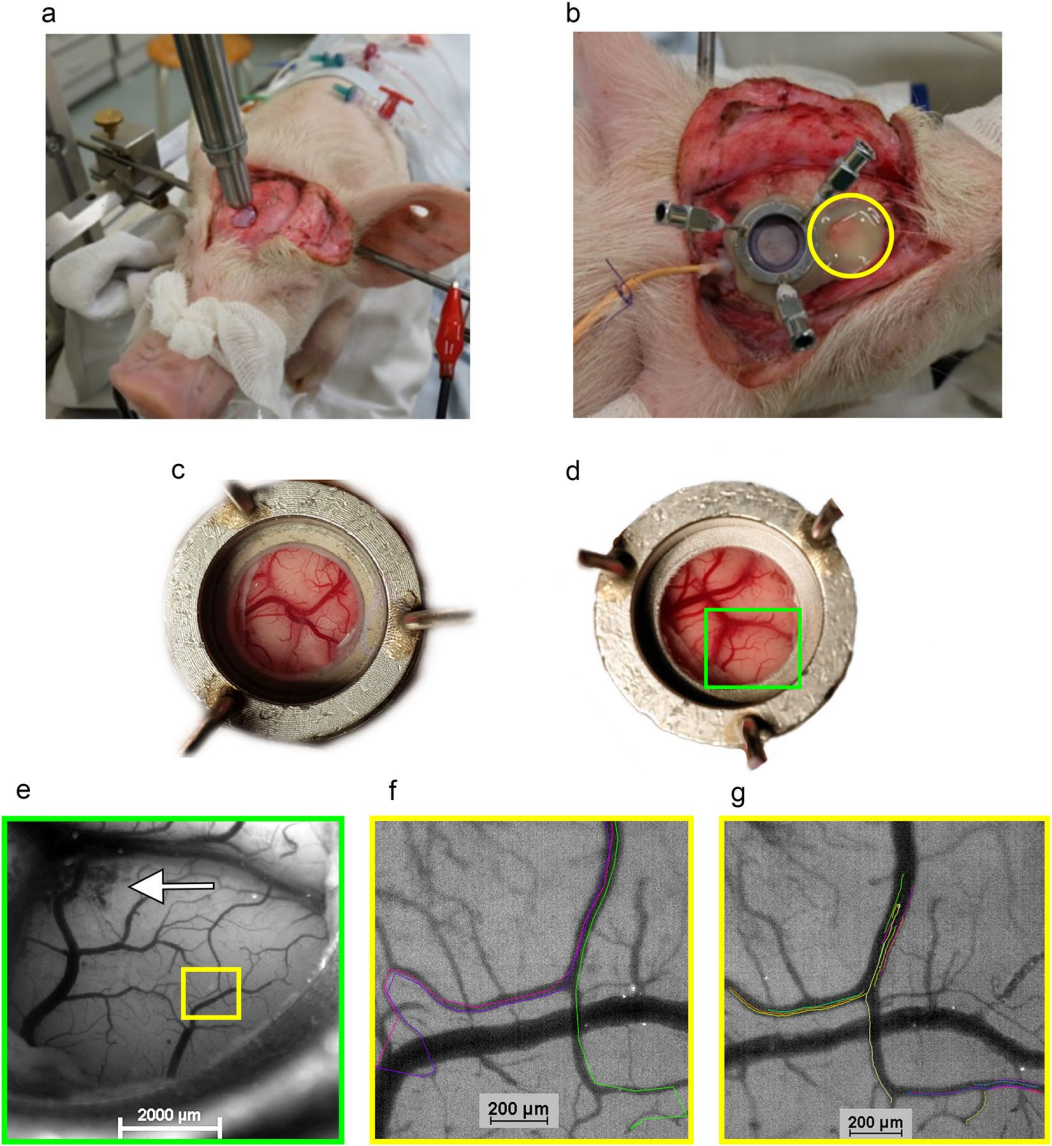
Illustration of *in vivo* RBC flux measurement in a porcine model of traumatic brain injury. (**a**) Controlled cortical impact (CCI) of 4 m/sec, 200 ms dwell time, 12 mm depth. A right frontal 1.5 cm diameter bone fragment was removed to expose the dura mater. (**b**) Placement of the closed cranial window immediately behind the contusion. Closure of the skull by replacement of the bone fragment and fixation using acrylic cement (yellow circle). (**c**) Placement of the closed cranial window over non-contused brain. (**d**) Placement of the closed cranial window directly over the contusion. Hyperemia and some gyral edema can be seen. (**e**) Zoom-in view of the green box region in (**d)**. The white arrow indicates cerebral microbleeds secondary to the CCI. (**f**) Zoom-in view of the yellow box region in (**e)** demonstrating individual RBC tracks superimposed on the original image in different colors at baseline cerebral perfusion pressure (CPP). (**g**) Zoom-in view of the yellow box region in **e** demonstrating individual RBC tracks superimposed on the original image in different colors and pial arteriolar vasodilation during balloon-induced hypotension with CPP of 35 mmHg.

We explored the feasibility of measurement of RBC flux using our method in a porcine controlled cortical impact model of traumatic brain injury (CCI of 4 m/sec, 200 ms dwell time, 12 mm depth). Figure 5 illustrates the CCI and placement of the closed cranial window immediately behind the contusion or directly over the contusion. In two animals the closed cranial window was implanted immediately behind the cortical contusion. In one animal the closed cranial window was implanted directly over the cortical contusion. The model was able to measure pial arteriolar RBC flux immediately behind and even over the cortical contusion 2 to 3 hours after the CCI without interference of TBI-induced artifacts. We demonstrated that our method can be used to study pial arteriolar RBC flux in a translational swine model of traumatic brain injury.

## Discussion

We developed a method for direct intravital measurement of pial arteriolar RBC flux in a porcine model allowing the study of CBF in physiological and pathophysiological conditions.

The main advantage of the present porcine pial arteriolar RBC flux method is the combination of vessel diameter and RBC velocity to provide a complete description of blood flow in individual vessels in a large animal model. In rodents the study of single vessel cerebral blood flow has led to significant advancements in the understanding of normal physiology and pathophysiology^9,10,17^. However, important differences exist between rodents and humans in terms of general physiology, brain structure, and function. A small animal size makes rigorous measurement of important physiological variables difficult to nearly impossible^14^. In large animal models, the study of cerebral blood flow on the level of individual vessels so far has been mainly limited to vessel diameter measurements as a surrogate of flow^24,25^. However, vessel diameter has been proven to be an incomplete measure of flow^10,11^.

We were able to validate pial arteriolar flow changes in the current model using regional cortical laser Doppler flow during non-pharmacological arterial blood pressure manipulation. LDF quantitatively measures changes in regional CBF^18–22^. Pial arteriolar RBC flux measured through the cranial window yielded similar results as nearby regional LDF measurements. The main determinant of LDF changes were changes in RBC velocity. The ability of the porcine pial arteriolar RBC flux method to detect the limits of cerebrovascular autoregulation was validated by comparing the pial arteriolar flow limits to the limits estimated by LDF and brain parenchymal oxygen tension during non-pharmacological manipulation of arterial blood pressure. Our results are consistent with previous studies using LDF to determine the lower and upper limits of autoregulation in a porcine model with blood pressure manipulation. Based on LDF measurements, the mean lower limit of autoregulation was at a CPP of 36.9 ± 10.1 mmHg in our experiments versus 36.2 ± 10.5 mmHg reported by Zeiler *et al*. and the mean upper limit of autoregulation was at a CPP of 101.1 ± 17.2 mmHg versus a median CPP of 102 mmHg (IQR 97–109) reported by Pesek *et al*.^26,27^. A very good agreement between the lower and upper limits of cerebrovascular autoregulation was found between pial arteriolar RBC flux, LDF, and brain parenchymal oxygen tension methods.

We demonstrated that the combination of vessel diameter and RBC velocity to calculate RBC flux in individual vessels remained effective in a porcine model of traumatic brain injury. Cortical impact has been demonstrated to reproducibly impair autoregulation capacity as measured by pial arteriolar diameter and LDF changes^28–30^.

We conclude that the porcine pial arteriolar RBC flux method provides a reliable method for individual pial arteriolar blood flow studies, allowing for in-depth investigation of the mechanisms underlying efficient and deficient CAR. Therefore, the detailed single arteriole blood flow measurement method in a large animal model has the potential to close the translational gap between experimental and clinical work on CAR.

## Materials and Methods

### Ethical considerations

All animal care and procedures were approved by the Ethical Committee for Animal Experimentation at the KU Leuven University (P105-2015) in compliance with the Belgian Royal Decree (29 May 2013) and European Directive 2010/63/EU on the protection of animals used for scientific purposes. All animal procedures were conducted under veterinarian supervision according to the guidelines imposed by the Ethical Committee.

### Animals and anesthesia

Twenty 6-week-old male piglets (domestic swine, Zootechnical Center at the KU Leuven University) were studied for measurement of the hemodynamic response and limits of CAR in pial arterioles and validation using laser Doppler flowmetry and brain tissue oxygenation measurements. Additionally, the feasibility of measurement of the pial arteriolar hemodynamic response in a TBI model was studied in three 6-week-old piglets. Animals were pre-medicated with an intramuscular dose of tiletamine/zolazepam 4 mg/kg (Zoletil® VIRBAC) and xylazine hydrochloride 1.6 mg/kg (Xylazine® V.M.D.). Vascular access was obtained via an ear vein. All animals were pre-oxygenated with 100% oxygen. Endotracheal intubation was provided with a cuffed endotracheal tube (Mallinckrodt®) after a bolus of propofol (Diprivan® AstraZeneca) (2 mg/kg) and fentanyl (Janssen-Cilag) (2 µg/kg) in prone position. After endotracheal intubation, oxygen was reduced to 40% mixed with air. Anesthesia was maintained with intravenous infusion of propofol (2–4 mg/kg/h), midazolam (B. Braun) (0.3–0.7 mg/kg/h) and fentanyl (2 µg/kg/h -adjusted to response to painful stimuli up to 20 µg/kg/h) all by continuous infusion until the end of the experiment. A pancuronium (Inresa) continuous infusion (0.3 mg/kg/h) was started before placement of the stereotactic frame and maintained until the end of the experiment. No inhalation anesthetics were used during any stage of the experiment. Ventilation was accomplished with a volume-controlled ventilator (Dräger Cato® Dräger) delivering tidal volumes of 10–15 ml/kg at a respiratory rate of 20–26/min adjusted to maintain an end-tidal carbon dioxide (CO2) tension of 40 mmHg verified by arterial blood gas sampling. At the end of the experiment the animal was euthanized with sodium pentobarbital (Euthasol® Kela Veterinaria).

### Functional CAR challenge using controlled non-pharmacological blood pressure manipulation

Because pharmacologic agents such as catecholamines or vasodilators could also affect the pial arteriolar response, this way confounding the results of the experiments, the CPP was manipulated using non-pharmacological methods. Two series of experiments were performed, a hypertensive and a hypotensive group, with 10 animals in each group. A sample size of 10 per group will yield statistically significant results at the p = 0.05 level with a power of 0.9 (one-tailed paired t-test). Progressive hyper- or hypotension was induced, depending on placement of a balloon catheter in the aorta or inferior vena cava, as described below. The balloon catheter was gradually inflated using a syringe pump over a period of 2 to 3 hours by infusion of saline. Maximum inflated balloon diameter was 13 mm after infusion of 1.5 mL saline. The infusion rate of the balloon ranged from 0.1–0.3 mL/hour and was adjusted to the arterial blood pressure response.

#### Surgical procedure

The left femoral artery was cannulated in supine position for placement of a pressure and blood gas monitoring line that was advanced to the thoracic aorta 5 cm above the diaphragm. In the hypertensive group the right femoral artery was cannulated for placement of a 5Fr balloon occlusion catheter (LeMaitre embolectomy catheter® 1601-54, LeMaitre Vascular). The balloon catheter was advanced into the thoracic aorta at the level of the diaphragm. In the hypotensive group the left femoral vein was cannulated for placement of a 5Fr balloon occlusion catheter that was advanced to the inferior vena cava at the level of the diaphragm. Heparin continuous infusion intravenous infusion was started upon catheters insertion (50 IU/kg/h). Animals were subsequently positioned in a custom made stereotactic frame, in prone position for cranial surgery. After a midline scalp incision and removal of the periosteum the surface of the right side of the skull was exposed. Two 3–4 mm burr holes were made over the right hemisphere behind the coronal suture to allow placement of intracranial pressure (ICP) and Laser Doppler flow (LDF) probes. After perforation of the dura an intraparenchymal probe for continuous measurement of ICP, brain temperature and brain tissue oxygen pressure (PbtO_2_) (Neurovent^®^-PTO, Raumedic AG Muenchenberg, Germany) was placed. A 3.5 mm diameter LDF probe was placed in contact with the dura (Moor VMS-LDF1 with VP14-CBF probe, Moor Instruments, Devon, UK) hereby avoiding large dural or pial vessels. The cranial window was placed according to the technique described by Levasseur *et al*. and Busija *et al*.^31,32^. A right frontal craniotomy was made anterior to the coronal suture using a high-speed drill, continuously cooled with cold saline. Meticulous hemostasis was performed of bone edges using bone wax. The dura was elevated, cut and removed under optical magnification to avoid damaging the underlying structures. A cranial window was placed over the craniotomy and cemented with dental acrylic cement (G-CEM LinkAce®, GC Europe). The cranial window consists of a stainless-steel ring fitted with 3 injection ports and a central 15 mm diameter opening sealed with a coverslip glass using acrylic glue ﻿(Histoacryl®, B. Braun, Germany) to prevent cerebrospinal fluid leakage. The space under window was filled with artificial cerebrospinal fluid using the injection ports (NaCl 132 mM/L, KCl 3.0 mM/L, MgCl_2_ 1.5 mM/L, CaCl_2_ 1.5 mM/L, urea 6.6 mM/L, glucose 3.7 mM/L, NaHCO_3_ 24.6 mM/L warmed to 37 °C and equilibrated with 6% O_2_ and 6% CO_2_ in N_2_ to a pH 7.35–7.45, pCO_2_ 40–42 mmHg and pO_2_ 42–50 mmHg). Injection ports were closed by 3-way stopcocks and Luer lock caps. Piglets were allowed to recover for 2 hours after cranial window placement.

The balloon catheter was gradually inflated using a syringe pump over a period of 2 to 3 hours. In the hypertensive group, a progressive increase in arterial blood pressure was induced by gradual inflation of the balloon catheter in the abdominal aorta, increasing cardiac output to the remaining circulation. In the hypotensive group a progressive decrease in arterial blood pressure was induced by slowly reducing systemic venous return. Total duration of the experimental procedures ranged from 7 to 9 hours.

#### Physiological monitoring and data storage

ABP, ICP, PbtO_2_, brain temperature and LDF signals were monitored continuously. Heart rate was monitored simultaneously by a 3 lead ECG and by arterial pulse wave analysis. Blood oxygen level was monitored using a pulse oximeter and kept at 99–100% during the entire experiment. Inspired and expired concentrations of CO_2_ and oxygen were monitored with a gas analyzer (Phillips M1026B, Philips Medical Systems, Netherlands). Arterial blood partial pressure of carbon dioxide (paCO_2_) was sampled for verification of continuously monitored end-tidal CO_2_. pH was kept in normal range 7.35–7.45, PaO_2_ at 200 mmHg and paCO_2_ at 38 mmHg. Rectal temperature was maintained at 38–39 °C by a warming mattress and blankets. Continuously monitored signals were stored using ICM+ software (Cambridge University, Cambridge, United Kingdom). ABP, ICP and LDF signals were sampled at 250 Hz. CPP was calculated as the difference between ABP and ICP.

### Fluorescent labeling of red blood cells

RBC’s were labeled with carboxyfluorescein diacetate succinimidyl ester (CFSE) (Invitrogen/Molecular Probes). CFSE is a membrane-permeable esterified cytoplasmatic dye commonly used for staining of viable cells without compromising their functional properties^33^. CFSE has an overlap of the excitation and emission spectrum with the autofluorescent biomolecules in brain tissue. Selective fluorescent filter illumination produces a negative contrast delineation of pial vessels^34^.

A total of 2 × 20 mL blood was sampled from the arterial line (estimated 4–7% of total blood volume for animals weighing 8–12 kg)^35^ in EDTA tubes (Vacutainer®, BD Biosciences, San Jose, CA, USA) and centrifuged for 10 min at 500 × g. Blood withdrawal was performed in two steps to minimize systemic effects (second blood withdrawal was performed immediately before injection of the first batch of labeled RBC’s). RBC’s were incubated in 40 μM CFDA-SE diluted in phosphate-buffered saline (PBS) for 15 min at 38 °C with periodic inversion to ensure uniform labeling. After incubation, RBC’s were centrifuged to remove unbound CDSE dye and washed twice with warm PBS at 38 °C. Washed and labeled RBC’s were resuspended in warm PBS up to their original volume of 2 × 20 mL for slow injection through the arterial line over 10 minutes.

### Cranial window *in vivo* imaging: microscope, camera settings, RBC velocity measurements, vessel diameter measurements

Pial vessels were observed through the cranial window using an epifluorescence microscope (SMZ18 with P2-SHR Plan Apo 1×, Nikon), illuminated with a solid-state light engine (SOLA SM2, Lumencor), and captured with a high-speed digital CMOS camera (Orca Flash 4.0 V2, Hamamatsu) controlled by NIS-Elements software (Nikon). A green fluorescent filter (P2-EFL GFP-B Filter Cube 470–535 nm, Nikon) was used. Images were acquired at 170–200 frames per second and digitally stored for offline analysis. Minimal requirements for a high-speed camera were determined. Since no direct data on pial arteriolar RBC velocity was available we extrapolated data from the pig coronary artery RBC velocity: for arterioles ranging from 30 μm to 140 μm a mean velocity of 54 mm/s and 27 mm/s respectively is described^36^. To be able to obtain 2–4 images per cell at a maximum speed of 60 mm/s a minimal framerate of 100–200 fps is necessary. With our setup arterioles up to 20–30 μm can be visualized.

RBC velocity was determined by threshold pixel intensity detection of fluorescent RBCs to create a binary object. An individual movement track was created and average path speed was calculated for each track. Per arteriole an overall average of RBC path speeds was calculated for each time point. Diameter measurements were performed for each time point. The microscope and camera system were calibrated to allow pixel to micrometer conversion. Diameter measurements were performed by pixel intensity thresholding between background and arteriole in the same region of interest per arteriole. NIS-Elements software (Nikon) allowed for automated measurements to be made based on minimum Feret diameter.

Under the assumption of steady-state laminar flow in a cylindrical vessel, RBC velocity and lumen diameter measured from a single vessel can be used to define the average volumetric flux F^→^, by:

$$F=V\ast A=V\ast \pi \ast {r}^{2}=\,V\ast \pi \ast {(D/2)}^{2}$$

where V is velocity, and A is the luminal cross-sectional area, r is the vessel radius, and D is the vessel diameter^16,17^.

For this study, we randomly selected up to 3 arterioles per cranial window experiment ranging from 20 to 140 μm for further analysis.

### Controlled cortical impact (CCI) model of traumatic brain injury

The feasibility of measurement of the pial arteriolar hemodynamic response in a TBI model using a controlled cortical impact was studied in three 6-week-old piglets. A right frontal 1.5 cm diameter circular bone fragment was removed to expose the dura mater. A severe electromagnetic CCI of 4 m/sec, 200 ms dwell time, 12 mm depth (Pinpoint PCI3000 Precision Cortical Impactor, Hatteras Instruments) was delivered. The CCI was delivered over the intact dura mater. The CCI was followed by implantation of a closed cranial window. In two animals the closed cranial window was implanted immediately behind the cortical contusion and the skull was closed by replacement of the bone fragment fixated using acrylic cement. In one animal the closed cranial window was implanted directly over the cortical contusion. After implantation of the closed cranial window, fluorescent labeling of red blood cells was performed and *in vivo* measurement of RBC flux was performed (as described above).

### Statistical analysis

Analysis was performed using R statistical software (R Core Team 2016. R: A language and environment for statistical computing. R Foundation for Statistical Computing, Vienna, Austria. URL https://www.R-project.org/). The following packages were employed: ggplot2, dplyr and segmented.

Breakpoints in the relation between RBC flux, LDF flux and PbtO_2_ as measures of CBF, and CPP were used to determine the lower- and upper limits of autoregulation (LLA, ULA). Segmental linear regression was used to define breakpoints in the relation between pial arteriolar RBC flux, LDF flux and PbtO_2_ measurements and CPP in the same hemisphere. Normal distribution was assessed using Shapiro-Wilk at the level of significance 0.05. Paired samples t-test was used to assess statistical differences between breakpoints. CPP binning was used to compare LDF flux to changes in arteriolar diameter, RBC velocity and pial arteriolar RBC flux. CPP was divided into 5 mmHg bins and average measures were calculated for each CPP bin. The coefficient of determination (R^2^) for three linear regression models with LDF flux as dependent variable and pial arteriolar diameter or RBC velocity or RBC flux as independent variable using average measures for 5-mmHg CPP bins was calculated to describe the proportion of variance in LDF flux that is explained by the independent variables described above.

RBC flux, LDF flux, and PbtO_2_ breakpoints as determined by segmental linear regression were compared using two-way random intraclass correlation coefficients for absolute agreement.

## Supplementary information

Video 1 - Legend

Video 1

## Data Availability

The data that support the findings of this study are available from the corresponding author upon reasonable request.
