# Supplementary material for: Autoregulation assessment by direct visualisation of pial arterial blood flow in the piglet brain
Source: Sci Rep. 2019 Sep 16;9:13333. doi: 10.1038/s41598-019-50046-x (PMC6746693; doi:10.1038/s41598-019-50046-x)
Supplement: Supplementary file 1 — Video 1 - Legend [file 41598_2019_50046_MOESM1_ESM.docx]

**Title page with author affiliations and contact information**

**Autoregulation assessment by direct visualisation of pial arterial blood flow in the piglet brain.**

S.P. Klein*^1^, V. De Sloovere^2^, G. Meyfroidt^3^, B. Depreitere^1^

1. Department of Neurosurgery, University Hospitals Leuven, Leuven, Belgium

2. Department of Anesthesiology, University Hospitals Leuven, Leuven, Belgium

3. Department of Intensive Care Medicine, University Hospitals Leuven, Leuven, Belgium

* Corresponding author: Samuel P. Klein, e-mail: sam.klein@kuleuven.be, address: Department of Neurosurgery, University Hospitals Leuven, Herestraat 49, 3000 Leuven, Belgium, tel.: +32 16 34 42 90

**Supplementary information: Video 1**

**Video 1.** **Fluorescent red blood cells through a pial arteriole** (slowed down to 25% of the original speed)**.**
